# East Texas Medical Association, 2nd Regular Meeting

**Published:** 1887-01

**Authors:** J. E. Mayfield

**Affiliations:** Secretary, E. T. M. A.


### EAST TEXAS MEDICAL ASSOCIATION.

The second session of this organization was held in the town of Rusk, on the 4th and 5th of January, 1887. Ten new members were received, making the total membership thirty-five, about twenty of whom were in attendance.

The word *regular* implies the qualification for membership. No limit as to territory. No fees or dues required. Expenses defrayed by tax, per capita. All students and practitioners are invited, and the public is welcome to attend.

The fraternity at Rusk defrayed all expenses of the visiting members and extended other liberal courtesies, for which a vote of thanks was returned. Among the enjoyable social features of the occasion were an address of welcome by Judge J. J. Perkins, and a visit to the penitentiary under escort of Rev. Woolam and others.

Dr. T. Y. T. Jameson, of Rusk, was elected president. It was voted that the Association desired representation in the Texas State Medical Association.

Several interesting medical subjects were lengthily discussed, especially those of the original papers read, viz: "Forceps Delivery" by Dr. J. H. Barham; "Typho-Malarial Fever," etc. by Dr. J. E. Mayfield; "Malarial Hæmaturia" by Dr. J. A. Abney; "A Case of Congenital Urethral Stricture," by Dr. W. G. Jameson.

Papers for next session: Puerperal Fever and Antisepsis, (held over by Dr. J. O. Furlow) ;Placenta Praevia, by Dr. A. M. Denman; Version in Delivery, by Dr. G. M. Abney; Chronic Gastric Catarrh, by Dr. J. M. Brittain; Diseases of the Rectum, by Dr. J. O. Lowrie.

Next session meets at Timpson, first Tuesday in April, 1887, at 7 p. m.

Secretary was requested, by vote, to furnish synopsis of proceeding to DANIELS TEXAS MEDICAL JOURNAL for publication.

J. E. MAYFIELD,
Secretary, E. T. M. A.

[The papers mentioned, will appear in next number of the JOURNAL.—ED.]

PRACTICE FOR SALE.—We call attention to Dr. Gregg's card. A knowledge of Spanish not necessary, tho' Dr. G. says it can be acquired easily, in a short time.

